# Comparative Analysis of Transformer Architectures and Ensemble Methods for Automated Glaucoma Screening in Fundus Images from Portable Ophthalmoscopes

**DOI:** 10.3390/vision9040093

**Published:** 2025-11-03

**Authors:** Rodrigo Otávio Cantanhede Costa, Pedro Alexandre Ferreira França, Alexandre César Pinto Pessoa, Geraldo Braz Júnior, João Dallyson Sousa de Almeida, António Cunha

**Affiliations:** 1 Computer Science Department, Universidade Federal do Maranhão (UFMA), Campus do Bacanga, São Luís 65085-580, Brazil; pedro.franca@discente.ufma.br (P.A.F.F.); alexandre.pessoa@nca.ufma.br (A.C.P.P.); geraldo@nca.ufma.br (G.B.J.); jdallyson@nca.ufma.br (J.D.S.d.A.); 2 School of Science and Technology, University of Trás-os-Montes e Alto Douro, Quinta de Prados, 5000-801 Vila Real, Portugal; acunha@utad.pt; 3 ALGORITMI Research Centre, University of Minho, 4800-058 Guimarães, Portugal

**Keywords:** fundus image, glaucoma screening, deep learning, classification, transformer, ensemble methods

## Abstract

Deep learning for glaucoma screening often relies on high-resolution clinical images and convolutional neural networks (CNNs). However, these methods face significant performance drops when applied to noisy, low-resolution images from portable devices. To address this, our work investigates ensemble methods using multiple Transformer architectures for automated glaucoma detection in challenging scenarios. We use the Brazil Glaucoma (BrG) and private D-Eye datasets to assess model robustness. These datasets include images typical of smartphone-coupled ophthalmoscopes, which are often noisy and variable in quality. Four Transformer models—Swin-Tiny, ViT-Base, MobileViT-Small, and DeiT-Base—were trained and evaluated both individually and in ensembles. We evaluated the results at both image and patient levels to reflect clinical practice. The results show that, although performance drops on lower-quality images, ensemble combinations and patient-level aggregation significantly improve accuracy and sensitivity. We achieved up to 85% accuracy and an 84.2% F1-score on the D-Eye dataset, with a notable reduction in false negatives. Grad-CAM attention maps confirmed that Transformers identify anatomical regions relevant to diagnosis. These findings reinforce the potential of Transformer ensembles as an accessible solution for early glaucoma detection in populations with limited access to specialized equipment.

## 1. Introduction

Glaucoma is a progressive optic neuropathy that affects the optic nerve and is characterized by the degeneration of the retinal ganglion cells. If left untreated, it can result in irreversible vision loss. The main cause of this damage is high intraocular pressure (IOP), often caused by inadequate accumulation of aqueous humor; this increase in eye pressure exerts continuous compression on the optic nerve, leading to permanent damage [1]. In addition, glaucoma is related to non-modifiable risk factors, such as advanced age and genetic predispositions [2]. These elements increase vulnerability to the development of glaucoma throughout a patient’s life.

This vulnerability is reflected in alarming global figures, as glaucoma is the leading cause of irreversible blindness worldwide. In 2020, approximately 76 million people were affected by glaucoma, and projections for the prevalence of this disease, mainly due to population aging, tend to increase to about 112 million cases by 2040 [2]. The biggest challenge in combating glaucoma is its silent nature. Because it is often asymptomatic in its early stages, many patients receive a late diagnosis, when significant and irreversible damage to their vision has already occurred [1].

The diagnosis of glaucoma usually involves a comprehensive clinical examination, which includes measuring IOP, assessing the visual field, and imaging tests, such as optical coherence tomography (OCT) [3]. These procedures require specialized equipment and trained professionals to perform them, resulting in considerable inequality in overall eye care, especially in low- and middle-income countries [4]. This situation is exacerbated in remote and disadvantaged communities, such as indigenous populations and ethnic minorities, where the rate of visual impairment is significantly higher due to the difficulty of access [5,6]. To address this gap, portable ophthalmoscopes are emerging as a promising solution, offering benefits such as portability, reduced cost, ease of use in remote locations, and integration with telemedicine for initial examinations [7]. However, their effectiveness in detecting diseases still faces limitations due to the low resolution of retinal images and the need for interpretation by specialists [8].

Recent advances in artificial intelligence (AI), especially in the field of deep learning, have transformed medical image analysis, enabling automated diagnostics and screening that are accurate and scalable for a variety of diseases [9]. Convolutional neural networks (CNNs) have shown exceptional performance in identifying conditions such as diabetic retinopathy, age-related macular degeneration, and glaucoma using retinal images [10,11]. More recently, Transformer architectures have been adapted for computer vision applications, yielding significant improvements in capturing long-range dependencies and contextual information in images [12]. These innovations have great potential to enhance glaucoma detection in retinal images, particularly when combined with handheld imaging devices [13].

Despite remarkable progress in AI in ophthalmology, most existing studies rely on high-resolution retinal fundus images captured by fixed and expensive equipment, predominantly employing CNNs [14]. This dependency limits the applicability of automated glaucoma screening in resource-poor settings or where access to specialized equipment is restricted.

In this context, the present work proposes to investigate the applicability of ensemble methods based on multiple Transformer architectures for glaucoma classification from low-resolution retinographs acquired with portable ophthalmoscopes connected to smartphones. The combination of different models seeks to explore the complementarity between learned representations, aiming to evaluate their potential to improve the robustness and reliability of automated screening under adverse acquisition conditions.

Thus, this work seeks to contribute to the expansion of the use of Transformer architectures in challenging clinical settings, strengthening the early detection of glaucoma, especially in regions with limited access to specialized ophthalmological infrastructure, and consequently contributing to the reduction in the global burden of blindness caused by this disease.

We organized this work as follows: Section 2 presents a review of related work, highlighting the main characteristics and contributions in glaucoma detection using retinal images. Section 3 details the proposed methodology, covering the data used, image preprocessing, selected Transformer models, as well as training and evaluation procedures. Section 4 presents the results obtained, accompanied by a detailed analysis that includes a case study and a comparison with related works. Finally, Section 5 presents the conclusions of this study, highlighting its contributions to the automated detection of glaucoma in portable devices and indicating possible avenues for future work.

## 2. Related Works

In Nakahara et al. (2022) [15], the focus was on a deep classifier based on ResNet-34, applied to images obtained with the D-Eye lens attached to an iPhone 8. The model was trained exclusively with photographs captured by the Nonmyd WX-3D clinical camera, manufactured by Kowa Company, Ltd. in Nagoya, Japan, and tested both in that domain and on smartphone images. The results showed a decline in performance when migrating from a clinical camera to a smartphone, with the AUC decreasing from 98.9% to 84.2%. In the advanced glaucoma scenario, a relative improvement was observed on the smartphone (AUC of 90.0%), which is still lower than that of the clinical camera (99.3%). Thus, it was found that training restricted to clinical images does not guarantee high performance when migrating to mobile acquisitions, which also explains the impact of the domain change by introducing challenges related to the need for dilation, manual selection of the best video frame, decentering, and blurring.

In Neto et al. (2022) [16], architectures for classification (Xception, ResNet152V2, and Inception-ResNetV2) and segmentation (U-Net with Inception-ResNetV2/V3) trained on high-resolution public datasets (RIM-ONE r3 [17], DRISHTI-GS [18], and REFUGE [19]), with high performance in AUROC (∼0.94–0.96 in cross-validation and ∼0.91 in leave-one-out) and moderate to high sensitivities (∼0.77–0.86), accompanied by equally high specificities (∼0.92–0.93). The activation maps generated by Grad-CAM (Gradient-weighted Class Activation Mapping) [20] focused on the excavation and neuroretinal ring, in accordance with clinical practice. However, when the same strategy was applied to a private D-Eye dataset (347 images captured by a smartphone-mounted lens, 512 × 512 crops, consisting of 293 normal and 54 glaucomatous images), an acceptable AUROC (0.79–0.87) and high specificities (∼0.92–0.99) were maintained, but with substantially lower sensitivity (0.16–0.49), indicating greater difficulty in identifying true positive cases in this domain. In some samples, Grad-CAMs shifted the saliency to peripheral vessels, reinforcing the challenge of sensitivity in lower-quality and contrast mobile images.

In Bragança et al. (2022) [21], the public Brazil Glaucoma (BrG) dataset is presented, consisting of 2000 images captured without pupil dilation using an iPhone 6s attached to a Welch Allyn PanOptic 11820 portable ophthalmoscope (field of view ∼25°), manufactured by Welch Allyn, Inc. in Skaneateles Falls, NY, USA. The images were centered on the optic disc and cropped to ∼400 × 400 px. The study reports typical noises in this regime, such as low lighting, ambient light interference, and reflections from the device itself, in addition to providing masks of the disc and excavation. For classification, an ensemble of CNNs (DenseNet, MobileNet, InceptionV3, ResNet50v2, and ResNet101) was proposed, pre-trained on ImageNet [22], which achieved 90.5% accuracy and an AUC of 0.965 in 5-fold cross-validation. Thus, the work not only introduced a challenging public dataset but also established an important benchmark for evaluating classification models in low-resource settings, underscoring the need for robust approaches, such as an ensemble method, to handle variations in quality.

Fan et al. (2023) [23] compared the performance of a data-efficient Vision Transformer (DeiT) and ResNet-50, both trained on the OHTS dataset [24], consisting of 66,715 retinal photographs obtained by high-resolution clinical cameras. In tests performed on the OHTS itself, the two models showed similar AUROCs (0.82–0.91, depending on the reference label). The work also highlights DeiT’s attention maps, which focus more locally on the optic disc and neuroretinal ring, in contrast to ResNet’s more diffuse protrusions. Thus, it is evident that Transformers offer advantages in terms of generalization and interpretability, although the study remains limited to the high-resolution clinical domain, with controlled acquisition conditions and superior quality compared to smartphone-coupled devices.

Angara and Kim (2024) [25] evaluated multiple CNNs (VGGs, ResNets, and DenseNets) and an ensemble in two distinct domains: LAG [26], composed of high-resolution clinical images, and BrG. The authors observed a significant drop in performance when models trained only on LAG were tested on BrG, confirming the difficulty of generalization between domains. When the two datasets were combined in training, consistent gains were observed, with further improvement in performance achieved by the ensemble. The proposed model, trained on LAG + BrG, achieved Kappa values of 0.8771, an accuracy of 0.9385, a sensitivity of 0.9467, a specificity of 0.9305, and an AUC of 0.9841 in BrG. In this context, it was highlighted that the integration of datasets of different natures mitigates domain imbalance and reinforces the competitiveness of CNNs, even though generalization in smartphone images requires architectural and training strategies that consider typical degradations in focus, contrast, and field coverage.

Costa et al. (2025) [27], a previous work of ours, investigated the use of Transformers applied directly to the BrG dataset, exploring four different architectures (ViT, SwinV2, DeiT, and BEiT). The networks were evaluated individually and also in a probability ensemble. In 5-fold cross-validation, the combined model achieved stable metrics, with an average accuracy of 93.25%, an F1-score of 93.10%, and an AUC of 97.95%, surpassing the performance of CNNs previously reported in BrG and approaching the results obtained by convolutional ensembles. It is worth noting that self-attention enables the capture of both global and local relationships that are robust to variations in lighting and texture commonly found in smartphone images [28].

The reviewed literature reveals a growing use of deep learning for glaucoma screening. However, many of these efforts, such as the works of Fan et al. (2023) [23] and Angara and Kim (2024) [25], often rely on high-resolution datasets obtained by fixed and expensive clinical cameras. Although Fan et al. demonstrated the advantages of the Transformer in terms of generalization and interpretability, their study was limited to high-quality clinical images. Angara and Kim, in turn, utilized a CNN ensemble on smartphone images, but encountered challenges in generalization across different domains and the need to combine datasets of varying natures. In contrast, our research differs in that it focuses exclusively on low-resolution retinographs and variable capture conditions, acquired using portable ophthalmoscopes attached to smartphones, with the aim of greater direct applicability in resource-limited settings.

The challenges inherent in mobile device images have been highlighted by several studies. Nakahara et al. (2022) [15] observed a significant drop in performance when testing models trained on clinical images with smartphone photographs, highlighting the need for pupil dilation and manual frame selection as limitations. Similarly, Neto et al. (2022) [16] demonstrated that CNN models, trained on high-resolution public datasets and then applied to the D-Eye dataset, showed substantially low sensitivity, indicating great difficulty in identifying true positives in low-quality images. Our work addresses these fundamental limitations by incorporating challenging datasets such as D-Eye (along with BrG) directly into the training process, rather than simply migrating pre-trained models to this domain.

This research builds upon our previous work, Costa et al. (2025) [27], which explored Transformer ensembles for glaucoma classification, albeit only in the BrG dataset. The present study advances this by integrating the D-Eye dataset, enabling a more comprehensive evaluation of the robustness of the Transformer under various acquisition conditions, and by introducing patient-level evaluation for D-Eye, which simulates clinical practice more realistically. Comparatively, while Bragança et al. (2022) [21] proposed CNN ensembles for smartphone images, our work is distinguished by an investigative proposal of Transformer architectures in an ensemble context, exploring their capabilities to capture global and local relationships, which can be particularly advantageous in images with noise and variable characteristics.

## 3. Materials and Methods

**Figure 1:**
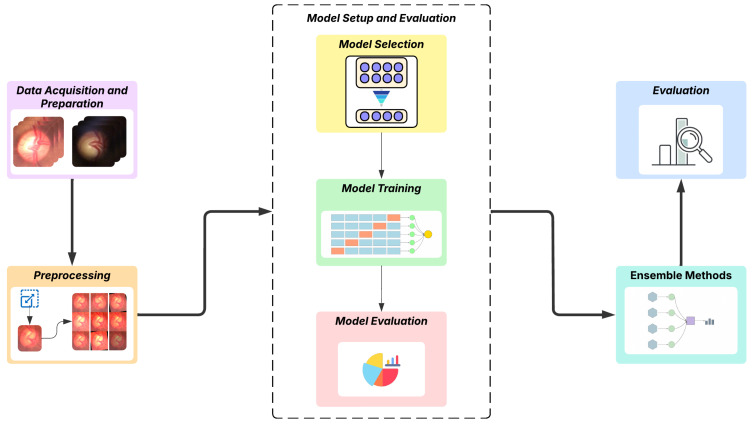
Flowchart of the applied methodology.

The proposed methodology of this work explores the application of deep learning, with an emphasis on the Transformer, for a computer vision task involving the binary classification of fundus images, distinguishing between glaucomatous and normal ones. The process begins with the acquisition of data from two datasets obtained using digital ophthalmoscopic devices connected to smartphones with different capture methods. In the next step, the images are preprocessed, which includes specific adaptations for the utilized models, as well as adjustments to improve training. Subsequently, we perform a preliminary analysis to select the most appropriate models, followed by training and evaluation of these models; the steps are organized in Section 3.3. After defining and configuring the models, we proceed to build the ensemble models and then evaluate the results using metrics specific to the classification task. Figure 1 visually summarizes the steps of the adopted methodology.

### 3.1. Data Acquisition and Preparation

For the development and evaluation of the method proposed in this work, two distinct datasets were used: the public Brazil Glaucoma (BrG) dataset and a private dataset that was provided for this work, obtained using the D-Eye device [29]. These datasets have specific characteristics and acquisition processes, which were considered when preparing the images for the subsequent stages.

The BrG dataset [21] is a public dataset that consists of 2000 fundus images from 1000 volunteers (500 patients with glaucoma and 500 without glaucoma), with images captured from both eyes. The images were captured with a Welch Allyn 11820 panoptic ophthalmoscope, coupled with an iPhone 6s smartphone, using the iExaminer app for acquisition, in clinical settings in Minas Gerais (Brazil). Both the ophthalmoscope and the acquisition software were developed by Welch Allyn, Inc. (Skaneateles Falls, NY, USA).

**Figure 2:**
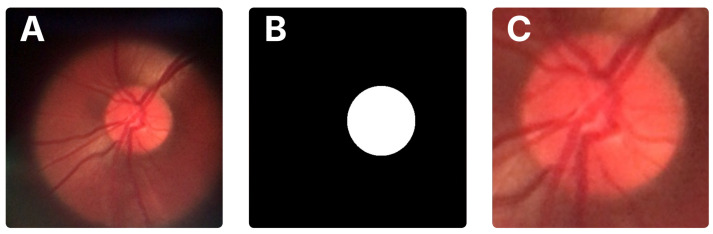
Data preparation process for the BrG dataset. (**A**) Original fundus image from the BrG dataset. (**B**) Binary mask corresponding to the optic disc, with the same resolution as the original image. (**C**) Final image after automatic cropping of the optic disc region, including a 15% margin.

A distinguishing feature of the BrG dataset is the availability of binary masks for the optic disc and cup, which facilitate precise localization of these structures. In this work, we used these masks to automatically crop the optic disc region from the original images. First, the optic disc pixels were identified in the mask to compute the smallest bounding box enclosing the area. These bounding box coordinates were then transferred to the original image to extract the corresponding region. To ensure the full disc and some surrounding context were included, the bounding box was expanded by 15% on all sides before cropping. Figure 2 illustrates the original image, the mask, and the resulting crop.

The D-Eye dataset is a private collection of images, obtained using a low-cost, portable eye imaging device called D-Eye, which consists of a lens attachment that fits directly onto a smartphone for retinal imaging. The D-Eye images were originally captured in video format (.mp4), necessitating a specialized sequence of processing steps to extract and select valid image frames.

The frames were initially selected by automatically extracting frames at regular intervals throughout the videos, which had an average duration of 15 s, followed by manual validation to ensure the quality and clear presence of the optic disc. The annotation of the optical disc region was performed manually and subsequently validated by a medical expert, who attested to the accuracy of the annotations made.

**Figure 3:**
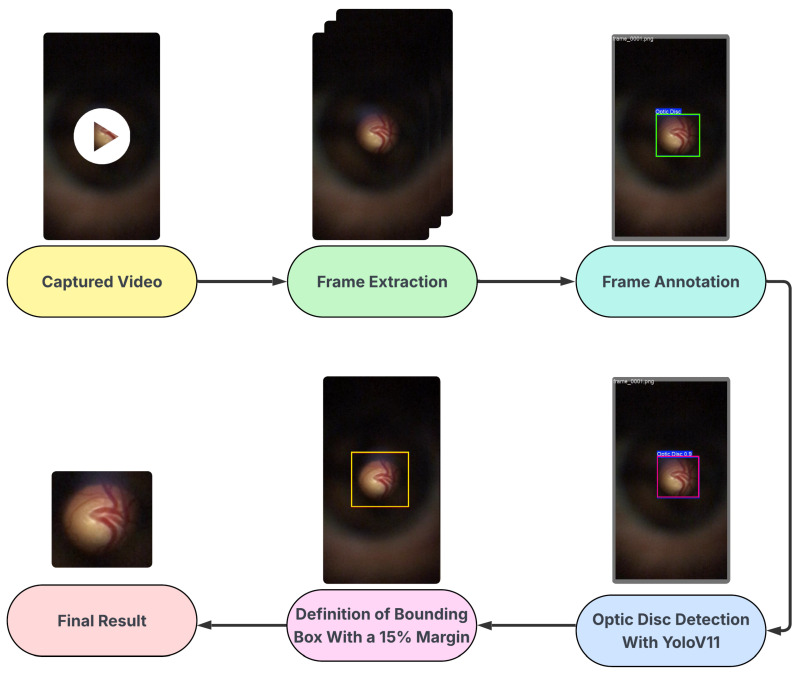
The data preprocessing pipeline for the D-Eye dataset, from video capture to final cropped optic disc images.

Subsequently, the manual annotation served as the basis for training a detection model using YOLO (You Only Look Once), specifically version YOLOv11 [30], which performed well in detecting optical discs in D-Eye images. This trained model was then used to automate the detection of the optic disc throughout the set, enabling the cropping of images with an additional 15% margin, similar to the procedure applied in the BrG dataset. Ultimately, the dataset comprises 518 images from 42 patients. The glaucomatous group consists of 243 images from 21 patients, with an average of 11.6 images per patient. The normal group contains 275 images from 21 control patients, with an average of 13.1 images per patient. The process described is represented in the flowchart shown in Figure 3.

**Figure 4:**
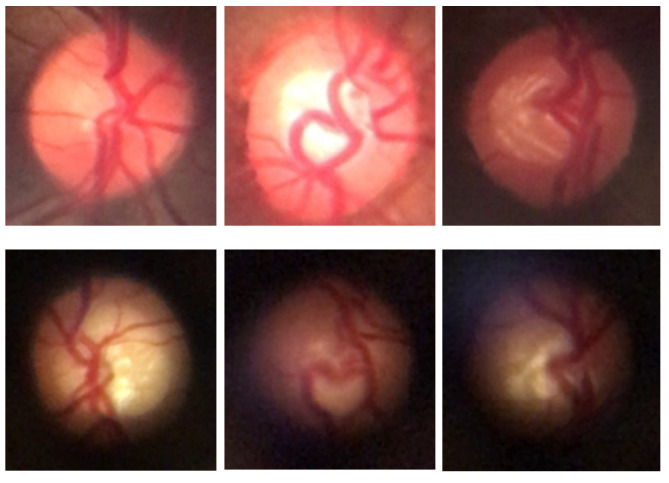
Sample fundus images from the datasets used in this work. The first row shows images from the BrG dataset, while the second row displays images from the D-Eye dataset.

For subsequent processes, the BrG and D-Eye datasets were merged to form a combined dataset, allowing for the exploration of image diversity across different devices and capture conditions. Figure 4 shows examples of fundus images from both datasets, highlighting the distinctive characteristics of each set, such as resolution, field of view, and image quality.

### 3.2. Preprocessing

To ensure the uniformity and suitability of the images for the neural network architectures used, all images from both datasets underwent an initial preprocessing step that included resizing and normalization before being fed into the networks. Resizing adjusted the images to the specific dimensions required by each model, with the most frequent resolution being 224 × 224 pixels, except for architectures requiring 256 × 256 pixel inputs. This standardization enabled batch processing and ensured stability during the training process.

To increase the robustness of the models and mitigate the risk of overfitting, a data augmentation scheme was adopted in the training set. For this, we used the Albumentations library [31], widely recognized for its efficiency and flexibility in image processing. Geometric transformations, such as horizontal inversion and small rotations, were applied so as not to compromise the relevant anatomical features. In addition, subtle adjustments were made to lighting, contrast, and colors, including the application of techniques such as local contrast enhancement using CLAHE (Contrast Limited Adaptive Histogram Equalization) and Gaussian blur to simulate natural variations in image quality. These transformations increase the variability of the training dataset, helping the model to better generalize to different visual conditions [32].

### 3.3. Model Setup and Evaluation

This stage encompasses procedures related to selecting Transformer models, network training, and performance evaluation in the task of classifying retinal images.

#### 3.3.1. Model Selection

To select the most suitable models for the classification task in this work, eight Transformer-based architectures were evaluated using the combined dataset from the two datasets used in this study. Training was performed using a hold-out scheme, where 70% of the data was allocated for training, and the remaining 30% was divided equally between validation and testing. This process was performed for 10 epochs, allowing for a quick and comparative analysis of performance. Table 1 shows the evaluated architectures, as well as the input dimensions used in each model.

For the subsequent steps, four models were selected: Swin-Tiny, ViT-Base, MobileViT-Small, and DeiT-Base. The choice took into account a balance between observed performance, efficiency, and architectural diversity. Details of the performance results that informed this selection, as well as a more in-depth discussion of the criteria adopted, are presented in Section 4.

#### 3.3.2. Model Training

The models were initialized from pre-trained weights in ImageNet [22], following a fine-tuning approach to adapt to the datasets used. Training was performed using k-fold cross-validation with 5 folds. For the BrG dataset, data was randomly split by image; for the D-Eye dataset, the data was split by patient, thus preserving the separation already specified in the dataset and preventing data leakage and bias in training.

Each fold was trained independently, meaning that model weights, optimizer states, and data splits were re-initialized for each fold to ensure no information was shared between them. This ensured reproducibility for training with the selected models. In training, we chose to use the AdamW optimizer [39], configured with a batch size of 32 and a total of 15 epochs. The learning rate was adjusted using a linear schedule with a warm-up period, and weight decay regularization was also applied. Additionally, the binary cross-entropy function was employed as the loss function for this binary classification task.

To take advantage of computational acceleration, training was performed with GPU support, using an NVIDIA RTX 3060 with 12 GB of VRAM. Furthermore, the implementation was based on the Pytorch framework (version 2.5.1) [40], using HuggingFace [41] to manage the models and the training process, and sklearn [42] to split the data into folds and calculate metrics.

#### 3.3.3. Model Evaluation

The evaluation of the models was conducted based on the predictions obtained in the validation sets defined during the k-fold cross-validation process. Considering that the training was performed using combined data from the BrG and D-Eye datasets, the performance analysis included both joint evaluation and separate evaluations for each dataset, respecting the specific structure of each dataset. Two distinct evaluation approaches were conducted during the validation stage.

In the first approach, evaluation was performed individually for both datasets; that is, each image in the validation set was treated as an independent sample for calculating the metrics. This means that the predictions are compared directly with the labels image by image, generating the metrics for the combined dataset and also for each dataset separately, always considering the images individually.

The second approach provides an assessment aligned with clinical practice, particularly for the D-Eye dataset, where diagnoses are typically consolidated based on the analysis of all patient images. Therefore, for BrG, which does not provide patient-level grouping, the assessment remained at the image level. For the D-Eye dataset, predictions were aggregated at the patient level during the evaluation, calculating the average of the predicted probabilities for all images of that patient in the validation set. The final prediction for each patient was then obtained by averaging these values, enabling a single and more representative classification for the individual.

**Figure 5:**
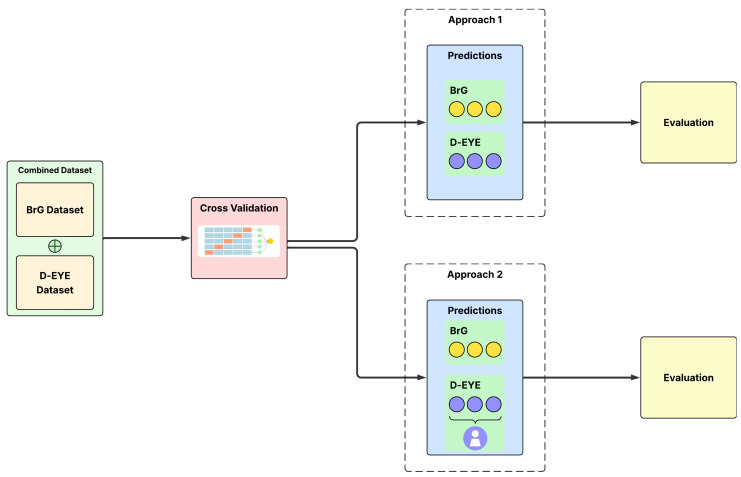
Illustration of image-level and patient-level evaluation approaches used in this study.

This dual evaluation strategy enables the model’s performance to be analyzed at both the granularity of the isolated image and at a more realistic and clinical level, particularly for the D-Eye dataset, where multiple images per patient are evaluated together to obtain a single diagnosis. The schematic representation of these two evaluation approaches is illustrated in Figure 5.

### 3.4. Ensemble Methods

The ensemble technique aims to combine the predictions of multiple individual models to enhance the performance and robustness of classification. In this work, we evaluate three different methods for aggregating the predictions of the four models trained in each fold of cross-validation, as described in Section 3.3.2. Table 2 provides a summary of the three methods evaluated, highlighting the basic aggregation principle for each.

In each fold, an ensemble model is constructed based on the aggregated predictions of the individual models, resulting in five distinct ensembles per method (one per fold). Subsequently, performance metrics are calculated for each ensemble, and then the mean and standard deviation are computed across the five folds, providing a robust and statistically consistent evaluation for each ensemble method.

The three ensemble methods were evaluated using both individual analysis of each image and aggregation of predictions at the patient level, as described in Section 3.3.3. This approach allows for a comprehensive comparison of the methods, considering both detailed performance at the image level and consistency and clinical relevance when decisions are consolidated by the patient for the D-Eye dataset.

### 3.5. Evaluation

To evaluate the proposed models, traditional classification metrics from supervised learning were used. Accuracy measures overall correctness, precision gauges the ratio of true positives among positive predictions, recall assesses the ability to identify all actual positives, specificity evaluates the correct identification of negatives, and the F1-score balances precision and recall. These metrics provide a comprehensive view of the models’ ability to correctly identify the classes of interest and consider different performance aspects.

In summary, these metrics are defined based on the values of true positive (TP), true negative (TN), false positive (FP), and false negative (FN), according to the following equations:

(1) $$\mathrm{Accuracy}=\frac{TP+TN}{TP+TN+FP+FN},$$

(2) $$\mathrm{Precision}=\frac{TP}{TP+FP},$$

(3) $$\mathrm{Recall}=\frac{TP}{TP+FN},$$

(4) $$\mathrm{Specificity}=\frac{TN}{TN+FP},$$

(5) $$F1-\mathrm{Score}=2\times \frac{\mathrm{Precision}\times \mathrm{Recall}}{\mathrm{Precision}+\mathrm{Recall}}.$$

## 4. Results

This section presents the results of our work. To facilitate comparison, the highest metric values for each dataset are highlighted in bold in all subsequent tables.

Table 3 and Table 4 show the average performance and standard deviation of each model evaluated by cross-validation. Table 3 presents the results for the first evaluation approach, in which predictions are analyzed individually for each image. The results are also presented for the combined dataset and for each dataset, BrG and D-Eye, separately. In turn, Table 4 presents the same indicators for the second approach, that is, the patient-level evaluation for the D-Eye dataset, aggregating the predictions per patient, while the evaluation for BrG remains at the image level.

Among the individual models, DeiT-Base achieved the highest metrics in the image-level evaluation, particularly on the higher-quality BrG dataset. When shifting from image-level to patient-level evaluation for the D-Eye dataset, a notable performance boost was observed across all models. In this setting, ViT-Base emerged as the most robust model, delivering the best results with an accuracy of 83.1%.

In addition, Table 5 and Table 6 show the average performance and standard deviation obtained by the evaluated ensemble methods. Table 5 presents the results obtained considering the individual evaluation per image, covering the combined dataset and the BrG and D-Eye datasets, separately. Table 6 presents the performance of the same methods under evaluation, with aggregation per patient for D-Eye, and maintaining the evaluation per image for BrG.

**Figure 6:**
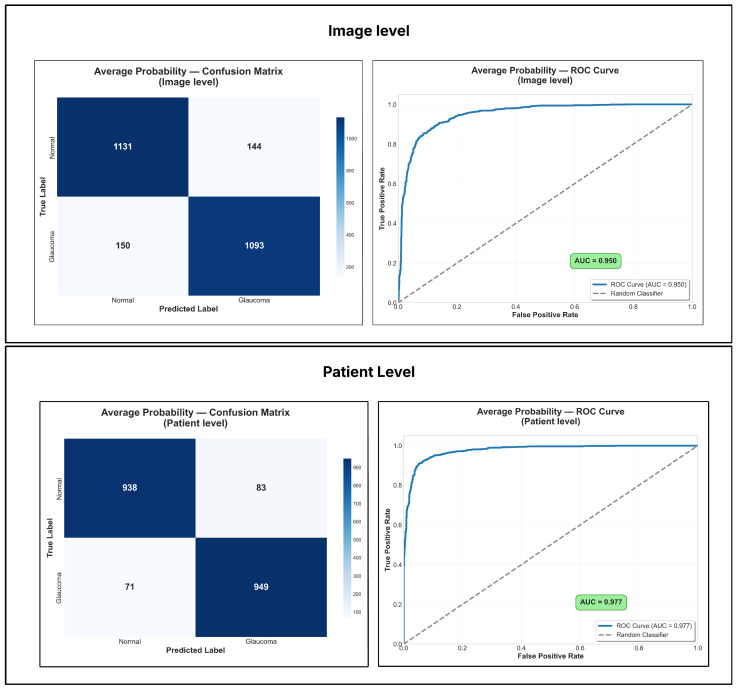
Confusion matrices and ROC curve with AUC value for the Average Probability ensemble method. The results are presented for both evaluation approaches: image-level and patient-level.

The ensemble methods further enhanced performance and robustness. For image-level evaluation, the Majority Vote strategy provided a strong balance across metrics, especially on the BrG dataset. However, more substantial improvements were seen after transitioning to patient-level aggregation for the D-Eye dataset. At this patient level, the Average Probability strategy proved most effective, achieving the highest recall (85.0%), a crucial metric for clinical screening scenarios. To complement this analysis, Figure 6 shows the confusion matrices and the ROC curve with AUC value for the Average Probability ensemble method, illustrating the classification result at the image and patient levels.

### 4.1. Discussion

The results presented in Table 7 show that, despite similar performances in the combined dataset, there are considerable variations in the BrG and D-Eye datasets, highlighting the importance of assessing the robustness of models in the face of data diversity. Swin-Tiny stood out for its consistency and overall better performance for the hold-out approach, especially in the D-Eye dataset, compared to the other models.

DeiT-Base and MobileViT-Small were selected for their computational efficiency and lower data requirements, as well as the good balance in obtained results, characteristics that are relevant for practical applications with limited resources. ViT-Base was, in turn, included to broaden the architectural diversity of the group. Limiting the selection to these four models—detailed in Section 3.3.1—aimed to balance architectural diversity and computational feasibility, ensuring a clear focus for the next stages of this study.

In the image evaluation, which treats each image individually (Table 3), we observed that all four models in the BrG dataset achieved high and consistent performance. DeiT-Base stood out a little more, achieving an average accuracy of 92.6%, precision of approximately 93.6%, and recall of 91.6%. These values indicate that it is effective both for identifying patients with glaucoma (high sensitivity) and for recognizing patients without the disease (high specificity of 93.6%). ViT-Base also performed robustly, achieving an accuracy of nearly 91.6% and an F1-score of approximately 91.8%, thereby reinforcing its classification ability in these images with better visual quality.

In the combined dataset, the models performed moderately, reflecting the mix of images of varying quality. For example, DeiT-Base achieved an average accuracy of around 88.4%, while ViT-Base obtained similar results, around 88.3%, indicating that the introduction of more challenging data from D-Eye impacts the overall average of the results, but without drastically degrading performance.

In contrast, in the D-Eye dataset, known for its lower quality images and greater variability, the results of all models suffered a considerable reduction. ViT-Base was the model that achieved the closest satisfactory performance in this scenario, with an average accuracy of approximately 75.6%, a recall of around 72.6%, and an F1-score of approximately 72.4%. Models such as MobileViT-Small presented lower values, with an average accuracy of 64.6% and an F1-score of 63.3%, indicating greater difficulty in handling these noisier and more unstable images. The high variability of the results in the metrics, as demonstrated by the high standard deviation (for example, ±17.9% in the accuracy of ViT-Base), highlights the heterogeneity and challenges related to D-Eye images.

When the evaluation was adapted to analyze the grouped prediction per patient in the D-Eye dataset, simulating a more realistic clinical practice, the results showed significant improvement (as shown in Table 4). The accuracy of ViT-Base increased from approximately 75.6% to 83.1%, and recall rose to 85%, indicating that the model becomes more reliable when considering multiple images from the same patient. DeiT-Base also showed improvement, achieving an average accuracy of around 80.3% and a sensitivity of 80%. In contrast, Swin-Tiny, which performed poorly at the image level (approximately 67.9% accuracy), maintained more modest results at the patient level, with approximately 71.1% accuracy and 70% recall. These data indicate that consolidating multiple images per patient helps smooth out isolated errors and makes classification more accurate and clinically meaningful for challenging datasets. In the BrG dataset, the evaluation per patient remained stable, as it remains at the image level.

When analyzing ensemble methods (Table 5 and Table 6), the trend toward increased robustness and performance becomes even clearer. In the evaluation using isolated images (Table 5), the Majority Vote strategy stood out for offering the best compromise between precision and recall, achieving 88.4% accuracy in the combined set, with precision above 90% and specificity above 91%. In the BrG dataset, this strategy achieved an accuracy of 92.6% and a significant precision of 93.9%, slightly surpassing the results obtained by individual models in this metric. However, in the D-Eye dataset, although the ensemble improves the metrics compared to the isolated models, performance is still limited, with an average accuracy of around 73.3% and a recall below 65%, in addition to high standard deviation values, which reinforces the difficulties imposed by low-resolution images, noise, and low lighting.

When the evaluation considered aggregation by patient (Table 6), the improvements were more significant in the D-Eye ensemble. The Average Probability ensemble stood out by achieving the best recall of approximately 85%, alongside an accuracy of about 85.2%. The Majority Vote ensemble showed comparable accuracy but a slightly lower recall, while the Max Confidence method also performed similarly, reinforcing the stability of the ensemble regardless of the utilized combination method. In the BrG dataset, all strategies performed similarly well, with an accuracy of approximately 92.5%, maintaining the same results as the image evaluation, as expected.

In summary, detailed analysis of metrics and different scenarios reinforces that, despite the progress brought about by the Transformer, the field of mobile imaging still poses considerable challenges, where isolated models have limitations in ensuring high accuracy and recall. Ensemble combination emerges as a strategy to compensate for individual errors, increasing system confidence and improving clinical applicability, especially when decisions are consolidated at the patient level.

### 4.2. Qualitative Analysis by Case Study

**Figure 7:**
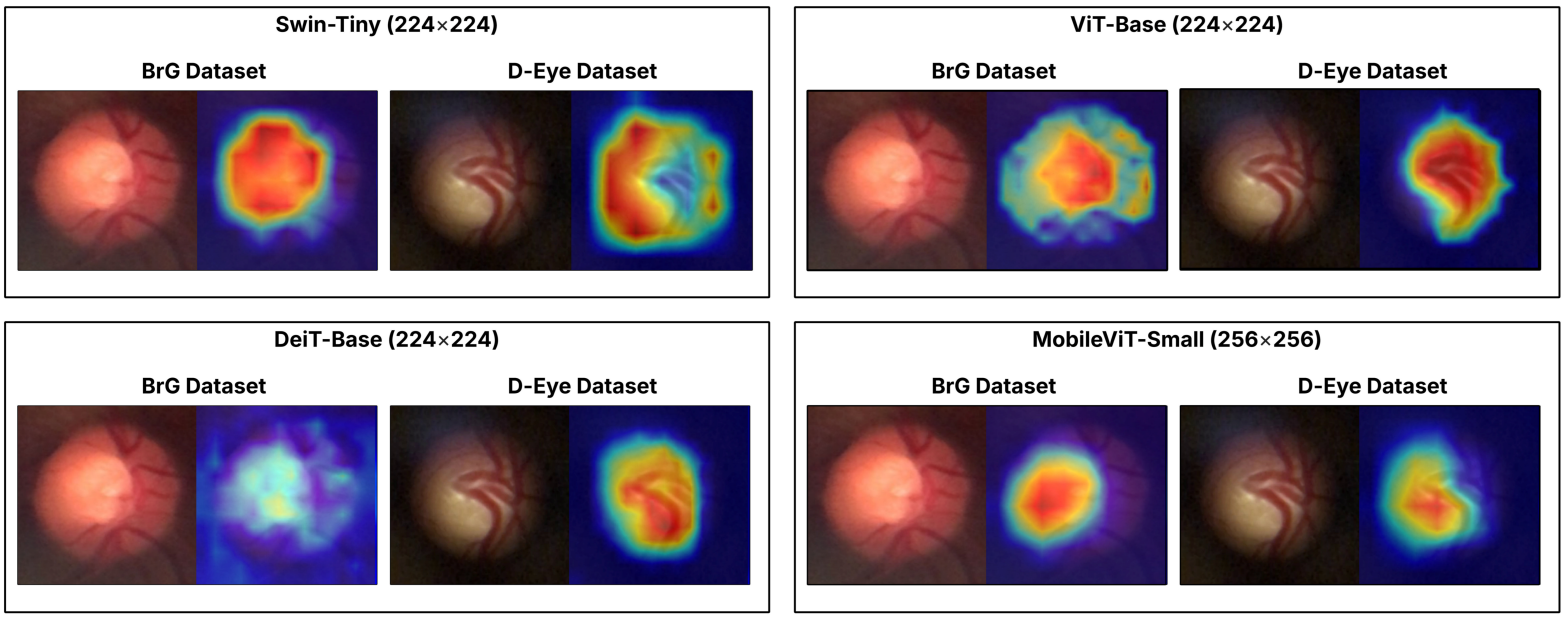
Grad-CAM activation maps of the models for glaucoma images from the BrG and D-Eye datasets. The heatmap illustrates the most important image regions for the models’ predictions, where the color scale ranges from blue (low importance) to red (high importance).

To qualitatively illustrate the performance of the models studied, we present a case study centered on Figure 7, which displays the activation maps generated by Grad-CAM [43] for an image from the BrG dataset and another from the D-Eye dataset, both of which contain glaucoma and were correctly classified by all evaluated models. This visualization highlights the differences in the attention and approach of Transformers when identifying regions relevant to disease diagnosis, showcasing the individual strengths and limitations of each architecture. This analysis reinforces the main objective of the work by demonstrating how the use of ensemble methods can combine these different perspectives, increasing the robustness and reliability of classification in challenging environments, with images from portable devices.

### 4.3. Comparative Analysis with Related Works

The Average Probability method was chosen for comparison with related works due to its superior performance in the D-Eye dataset, the most challenging in the analysis, with emphasis on high recall. Table 8 presents a comparison of the performance of the proposed model, using this ensemble, with previous studies on the BrG and D-Eye datasets, highlighting the competitiveness and advances achieved by the approach.

The comparative analysis shows that the proposed model, using the Average Probability ensemble, performs competitively in both the BrG and D-Eye datasets. For the BrG dataset, the metrics of the proposed model align with the results of recent studies, such as those by Costa et al. (2025) [27] and Angara and Kim (2024) [25], which present slightly higher or equivalent metrics.

In the D-Eye dataset, which represents a more difficult scenario due to lower-quality images and greater variability, the proposed model maintains competitive performance, with accuracy (∼85.2%) and sensitivity (∼85.0%) far superior to the values reported by Neto et al. (2022) [16], especially in recall, which practically doubles (from ∼49% to ∼85%). This is a significant advance, given the importance of sensitivity in diagnosis to minimize false negatives, which can lead to delays in treatment. It should be noted that the higher accuracy values with a low standard deviation and high specificity reported by Neto et al. are influenced by the significant class imbalance in their dataset, which consists of 347 images, with 293 normal and only 54 glaucomatous. The substantial improvement demonstrated here indicates that the use of the Transformer-based ensemble, alongside refined preprocessing and training, contributes to more robust detection even under adverse conditions.

Overall, the results indicate that the proposal is effective for images captured by mobile devices in various acquisition contexts, such as the BrG and D-Eye datasets, each with its own distinct characteristics. The balance between sensitivity and specificity, as evidenced in the metrics, reinforces the practical applicability of the method for glaucoma screening, especially in scenarios where mobile devices are used to expand access to diagnosis.

## 5. Conclusions

This study demonstrated the feasibility and effectiveness of using ensembles based on multiple Transformer architectures for automated glaucoma screening from retinal images acquired by portable ophthalmoscopes connected to smartphones. The combination of the BrG and D-Eye datasets enabled us to explore various acquisition conditions, including low-resolution images of varying quality, which are typical of mobile devices, thereby challenging traditional models based on high-quality clinical images.

The results indicate that, despite the limitations imposed by image variability and quality, especially in the D-Eye dataset, the application of Transformer ensembles promotes significant improvements in classification robustness and sensitivity, particularly when consolidating predictions at the patient level, which aligns with clinical practice. Additionally, the use of activation maps (Grad-CAM) demonstrated that Transformers can automatically identify anatomical regions relevant to diagnosis, thereby reinforcing the clinical interpretability of the system.

For future work, it is essential to evaluate the model’s generalization ability in a cross-dataset scenario, utilizing high-quality datasets to verify the adaptability of Transformers to various capture conditions. It is also recommended to experiment with different parameters and training methods, including specific data augmentation strategies for each dataset. Additionally, investing in robust improvements to image preprocessing can enhance the extraction of features crucial for classification, thereby further increasing the system’s accuracy.

Thus, this work reinforces the potential of Transformers to democratize access to early glaucoma diagnosis, contributing to the reduction in preventable blindness in populations with limited access to specialized equipment.

## Figures and Tables

**Table 1:** Pre-trained Transformer models with RGB color format.

Model	Input Size
ViT-Base [33]	224 × 224 × 3 (RGB)
ViT-Large [33]	224 × 224 × 3 (RGB)
DeiT-Base [34]	224 × 224 × 3 (RGB)
Swin-Tiny [35]	224 × 224 × 3 (RGB)
Swin-Base [35]	224 × 224 × 3 (RGB)
SwinV2-Tiny [36]	256 × 256 × 3 (RGB)
BEIT-Base [37]	224 × 224 × 3 (RGB)
MobileViT-Small [38]	256 × 256 × 3 (RGB)

**Table 2:** Description of the evaluated ensemble methods.

Method	Description
Simple Averaging	Selects the class with the highest Average Probability across all models.
Majority Voting	Selects the class that receives the most votes from the individual models.
Maximum Probability	Adopts the prediction from the model reporting the highest probability (confidence) for its class.

**Table 3:** Performance of individual Transformer models using image-level evaluation.

Model	Dataset	Accuracy	Precision	Recall	Specificity	F1-Score
DeiT-Base	Combined	0.8836±0.0320	0.8924±0.0389	0.8710±0.0335	0.8961±0.0442	0.8812±0.0302
	BrG	0.9260±0.0140	0.9357±0.0306	0.9160±0.0124	0.9360±0.0327	0.9254±0.0128
	D-Eye	0.7253±0.1599	0.7437±0.1974	0.6970±0.1370	0.7537±0.2214	0.7102±0.1404
MobileViT-Small	Combined	0.8520±0.0281	0.8448±0.0487	0.8618±0.0393	0.8441±0.0526	0.8520±0.0261
	BrG	0.9060±0.0186	0.8966±0.0160	0.9180±0.0291	0.8940±0.0175	0.9070±0.0192
	D-Eye	0.6463±0.1214	0.6799±0.2117	0.6338±0.0986	0.6711±0.2488	0.6327±0.0821
Swin-Tiny	Combined	0.8645±0.0338	0.8580±0.0328	0.8701±0.0466	0.8595±0.0338	0.8636±0.0345
	BrG	0.9140±0.0129	0.9072±0.0328	0.9240±0.0156	0.9040±0.0391	0.9151±0.0108
	D-Eye	0.6792±0.1707	0.6744±0.2119	0.6571±0.2229	0.7042±0.1878	0.6495±0.1946
ViT-Base	Combined	0.8830±0.0408	0.8809±0.0552	0.8861±0.0510	0.8804±0.0652	0.8823±0.0399
	BrG	0.9165±0.0232	0.9100±0.0392	0.9260±0.0096	0.9070±0.0441	0.9176±0.0214
	D-Eye	0.7565±0.1799	0.7510±0.1908	0.7264±0.2493	0.7900±0.1812	0.7245±0.2177

**Table 4:** Performance of individual Transformer models using patient-level evaluation for the D-Eye dataset.

Model	Dataset	Accuracy	Precision	Recall	Specificity	F1-Score
DeiT-Base	Combined	0.9236±0.0138	0.9330±0.0303	0.9137±0.0157	0.9334±0.0323	0.9229±0.0128
	BrG	0.9260±0.0140	0.9357±0.0306	0.9160±0.0124	0.9360±0.0327	0.9254±0.0128
	D-Eye	0.8028±0.1910	0.8333±0.2357	0.8000±0.2092	0.8000±0.2739	0.8029±0.1847
MobileViT-Small	Combined	0.9020±0.0176	0.8913±0.0149	0.9157±0.0280	0.8883±0.0161	0.9032±0.0182
	BrG	0.9060±0.0186	0.8966±0.0160	0.9180±0.0291	0.8940±0.0175	0.9070±0.0192
	D-Eye	0.7083±0.1382	0.7476±0.2379	0.8000±0.2092	0.6300±0.3493	0.7302±0.1024
Swin-Tiny	Combined	0.9098±0.0115	0.9030±0.0293	0.9196±0.0168	0.9001±0.0352	0.9108±0.0096
	BrG	0.9140±0.0129	0.9072±0.0328	0.9240±0.0156	0.9040±0.0391	0.9151±0.0108
	D-Eye	0.7111±0.1761	0.6933±0.2127	0.7000±0.3260	0.7200±0.1891	0.6759±0.2459
ViT-Base	Combined	0.9147±0.0230	0.9082±0.0401	0.9245±0.0096	0.9050±0.0456	0.9159±0.0210
	BrG	0.9165±0.0232	0.9100±0.0392	0.9260±0.0096	0.9070±0.0441	0.9176±0.0214
	D-Eye	0.8306±0.1650	0.8167±0.1708	0.8500±0.2236	0.8100±0.2074	0.8243±0.1815

**Table 5:** Performance of ensemble methods using image-level evaluation.

Strategy	Dataset	Accuracy	Precision	Recall	Specificity	F1-Score
Average Probability	Combined	0.8837±0.0348	0.8858±0.0437	0.8800±0.0428	0.8880±0.0494	0.8822±0.0334
	BrG	0.9240±0.0189	0.9237±0.0281	0.9250±0.0106	0.9230±0.0299	0.9242±0.0181
	D-Eye	0.7332±0.1813	0.7411±0.2159	0.7043±0.2164	0.7713±0.2041	0.7075±0.2020
Majority Vote	Combined	0.8844±0.0369	0.9047±0.0400	0.8576±0.0469	0.9113±0.0400	0.8800±0.0374
	BrG	0.9260±0.0140	0.9387±0.0244	0.9120±0.0057	0.9400±0.0250	0.9251±0.0134
	D-Eye	0.7274±0.1771	0.7516±0.2070	0.6462±0.2289	0.8126±0.1548	0.6806±0.2069
Max Confidence	Combined	0.8837±0.0408	0.8852±0.0420	0.8798±0.0569	0.8878±0.0462	0.8817±0.0416
	BrG	0.9245±0.0185	0.9235±0.0352	0.9270±0.0045	0.9220±0.0382	0.9250±0.0170
	D-Eye	0.7322±0.1978	0.7198±0.2122	0.6943±0.2675	0.7755±0.1768	0.6927±0.2435

**Table 6:** Performance of ensemble methods using patient-level evaluation for the D-Eye dataset.

Strategy	Dataset	Accuracy	Precision	Recall	Specificity	F1-Score
Average Probability	Combined	0.9225±0.0169	0.9222±0.0268	0.9235±0.0074	0.9215±0.0290	0.9228±0.0160
	BrG	0.9240±0.0189	0.9237±0.0281	0.9250±0.0106	0.9230±0.0299	0.9242±0.0181
	D-Eye	0.8520±0.1588	0.8433±0.1507	0.8500±0.2236	0.8433±0.1507	0.8421±0.1829
Majority Vote	Combined	0.9240±0.0136	0.9369±0.0240	0.9098±0.0044	0.9382±0.0248	0.9230±0.0129
	BrG	0.9260±0.0140	0.9387±0.0244	0.9120±0.0057	0.9400±0.0250	0.9251±0.0134
	D-Eye	0.8270±0.1382	0.8433±0.1507	0.8000±0.2092	0.8433±0.1507	0.8135±0.1620
Max Confidence	Combined	0.9225±0.0169	0.9220±0.0338	0.9245±0.0082	0.9205±0.0370	0.9229±0.0154
	BrG	0.9245±0.0185	0.9235±0.0352	0.9270±0.0045	0.9220±0.0382	0.9250±0.0170
	D-Eye	0.8298±0.1784	0.8267±0.1673	0.8000±0.2739	0.8433±0.1507	0.8063±0.2192

**Table 7:** Preliminary performance analysis of Transformer architectures for model selection.

Model	Dataset	Accuracy	Precision	Recall	Specificity	F1-Score
ViT-Base	Combined	0.8595	0.8596	0.8500	0.8684	0.8547
	BRG	0.9033	0.9172	0.8867	0.9200	0.9017
	D-Eye	0.6714	0.6061	0.6667	0.6750	0.6349
ViT-Large	Combined	0.8432	0.8144	0.8778	0.8105	0.8449
	BRG	0.9100	0.9073	0.9133	0.9067	0.9103
	D-Eye	0.5571	0.4884	0.7000	0.4500	0.5753
DeiT-Base	Combined	0.8622	0.8486	0.8722	0.8526	0.8603
	BRG	0.9067	0.9122	0.9000	0.9133	0.9060
	D-Eye	0.6714	0.5946	0.7333	0.6250	0.6567
Swin-Tiny	Combined	**0.8757**	**0.8722**	0.8722	**0.8789**	**0.8722**
	BRG	0.9100	**0.9241**	0.8933	**0.9267**	0.9085
	D-Eye	**0.7286**	**0.6571**	**0.7667**	**0.7000**	**0.7077**
Swin-Base	Combined	0.8405	0.8270	0.8500	0.8316	0.8384
	BRG	0.8900	0.8980	0.8800	0.9000	0.8889
	D-Eye	0.6286	0.5526	0.7000	0.5750	0.6176
SwinV2-Tiny	Combined	0.8703	0.8511	**0.8889**	0.8526	0.8696
	BRG	**0.9133**	0.9079	**0.9200**	0.9067	**0.9139**
	D-Eye	0.6857	0.6111	0.7333	0.6500	0.6667
BeIT-Base	Combined	0.8514	0.8415	0.8556	0.8474	0.8485
	BRG	0.9000	0.9054	0.8933	0.9067	0.8993
	D-Eye	0.6429	0.5714	0.6667	0.6250	0.6154
MobileViT-Small	Combined	0.8595	0.8478	0.8667	0.8526	0.8571
	BRG	0.9000	0.8947	0.9067	0.8933	0.9007
	D-Eye	0.6857	0.6250	0.6667	0.7000	0.6452

**Table 8:** Comparison of the metrics of the proposed model with related works for the BrG and D-Eye databases.

Model	Accuracy	Recall	Precision	Specificity	F1-Score
**BrG Dataset**					
Bragança et al. (2022) [21]	0.9085	0.8500	0.9550	0.9600	0.8990
Angara et al. (2024) [25]	0.9385	0.9467	-	0.9305	-
Costa et al. (2025) [27]	0.9325±0.0157	0.9310±0.0193	0.9342±0.0207	0.9340±0.0224	0.9324±0.0156
Proposed Approach	0.9240 ± 0.0189	0.9250 ± 0.0106	0.9237 ± 0.0281	0.9230 ± 0.0299	0.9242 ± 0.0181
**D-Eye Dataset**					
Neto et al. (2022) [16]	0.8500±0.0400	0.4900±0.2200	-	0.9200±0.0800	0.4900±0.1000
Proposed Approach	0.8520±0.1588	0.8500±0.2236	0.8433±0.1507	0.8433±0.1507	0.8421±0.1829

## Data Availability

The original contributions presented in the study are included in the article. Further inquiries can be directed to the corresponding authors.
